# Polysomnographic characteristics of excessive daytime sleepiness in postmenopausal women with obstructive sleep apnea

**DOI:** 10.3389/frsle.2026.1853970

**Published:** 2026-08-25

**Authors:** Thanida Manitchotpisit, Pansak Sugkraroek, Charnsiri Segsarnviriya, Naricha Chirakalwasan, Mart Maiprasert

**Affiliations:** 1 Department of Anti-Aging and Regenerative Medicine, College of Integrative Medicine, Dhurakij Pundit University, Bangkok, Thailand; 2 Department of Otorhinolaryngology, Samitivej Thonburi Hospital, Bangkok, Thailand; 3 Division of Pulmonary and Critical Care Medicine, Department of Medicine, Faculty of Medicine, Chulalongkorn University, Bangkok, Thailand; 4 Excellence Center for Sleep Disorders, King Chulalongkorn Memorial Hospital, Thai Red Cross Society, Bangkok, Thailand

**Keywords:** Epworth sleepiness scale, excessive daytime sleepiness, obstructive sleep apnea, polysomnography, postmenopausal women

## Abstract

**Introduction:**

Excessive daytime sleepiness (EDS) in obstructive sleep apnea (OSA) is linked to adverse cardiometabolic and safety outcomes, but established correlates may not apply to postmenopausal women. Data on polysomnographic determinants of EDS in this group remain limited. We examined associations between polysomnographic parameters and EDS and identified key parameters that may help discriminate EDS in postmenopausal women with OSA.

**Methods:**

In this retrospective cross-sectional study, 216 postmenopausal women with OSA, confirmed by Level 1 polysomnography (PSG), were classified as having EDS based on the Epworth Sleepiness Scale (ESS 10; *n* = 110) or as non-EDS (ESS < 10; *n* = 106). Polysomnographic variables were compared, and multivariable logistic regression was used to build a polysomnographic multivariable model.

**Results:**

Women with EDS were younger and had higher BMI and neck circumference than those without EDS (*p* < 0.05). Higher respiratory disturbance index (RDI) values (total, supine, REM, and NREM) and lower minimum oxygen saturation were independently associated with EDS. A four-variable model (RDI, REM RDI, mean, and minimum oxygen saturation) showed moderate discrimination (AUROC 0.693).

**Discussion:**

EDS was more frequent among women with greater respiratory burden and worse nocturnal hypoxemia. Future studies using multidimensional data may improve model performance and clinical applicability.

## Introduction

OSA is a common sleep-related breathing disorder. In Thailand, OSA prevalence was estimated at 15.4% among men and 6.3% among women (Neruntarat and Chantapant, 2011). Recurrent obstruction of the upper airway during sleep is a defining feature of OSA. This process leads to intermittent hypoxia and sleep disruption, which are associated with EDS, cognitive and functional impairment, and increased cardiometabolic and all-cause mortality risk (Garbarino et al., 2018; Jordan et al., 2014). EDS is associated with poorer quality of life, impaired academic and work performance, and a higher risk of motor vehicle accidents (Garbarino et al., 2018; Jordan et al., 2014). Assessing EDS in patients with OSA is therefore essential, as its presence is linked to poorer prognosis, treatment-related decisions, and patient engagement. However, EDS does not occur in all patients with OSA (Ulander et al., 2022; Roure et al., 2008; Omobomi et al., 2019), and the determinants of sleepiness may differ across patient subgroups. In women, OSA is further influenced by sex-specific anatomical, hormonal, and psychosocial factors. Postmenopausal status is associated with increased OSA risk, weight gain, changes in fat distribution, and sleep disturbance, all of which may influence the expression of EDS (Perger et al., 2019; Mirer et al., 2017).

OSA and EDS can be approached within a data-driven stratification framework, classifying patients into clinically relevant subgroups to support individualized assessment (Garbarino et al., 2018; Bouloukaki et al., 2025). Most previous studies, conducted mainly in male or mixed-gender OSA cohorts, have identified several factors associated with an increased risk of EDS, including younger age, comorbidities, elevated body mass index (BMI), a higher arousal index, a higher apnea–hypopnea index (AHI), higher sleep efficiency, lower mean oxygen saturation, and lower nadir oxygen saturation. However, findings have been inconsistent, with some studies reporting significant associations between EDS and younger age, higher AHI, or lower mean oxygen saturation, whereas others have found no such relationships. Consequently, the associations between demographic characteristics, polysomnographic parameters, and EDS remain incompletely understood (Ulander et al., 2022; Roure et al., 2008; Omobomi et al., 2019; Sun et al., 2012; Mediano et al., 2007). However, postmenopausal women—who experience substantial hormonally mediated changes and a rising burden of OSA—remain underrepresented, and data on polysomnographic determinants of EDS in this group are notably limited (Perger et al., 2019; Geer and Hilbert, 2021).

Therefore, we aimed to examine the associations between polysomnographic parameters and EDS and to identify polysomnographic variables that may help discriminate postmenopausal women with OSA according to EDS status. We hypothesized that indices of sleep fragmentation and nocturnal hypoxemia would be independently associated with EDS. We further hypothesized that these polysomnographic measures would serve as clinically relevant markers to discriminate between women with and without EDS in this population.

## Material and methods

### Study design and setting

This retrospective cross-sectional study examined the association between polysomnographic parameters and EDS in postmenopausal women with OSA, using data from a single-center, hospital-based sleep laboratory registry at Samitivej Thonburi Hospital, Bangkok, Thailand. Clinical and polysomnographic data were retrieved from medical records for postmenopausal women who underwent in-laboratory Level 1 PSG, including both full-night and split-night studies, between September 2018 and June 2025.

### Inclusion and exclusion criteria

Eligible participants were postmenopausal women (defined as at least 1 year since the last spontaneous menstruation, without surgical menopause) with a diagnosis of OSA according to the International Classification of Sleep Disorders, Third Edition, Text Revision (ICSD-3-TR), confirmed by Level 1 PSG, including both full-night and split-night studies with a minimum total sleep time of 2 h during the diagnostic portion for the split-night studies. Participants whose PSG demonstrated central sleep apnea during the scoring process were not included in the study. Participants were required to have completed the Thai version of the ESS (Banhiran et al., 2011) on the same day as the PSG as part of their diagnostic evaluation and before initiation of any therapeutic intervention.

Exclusion criteria included concomitant physical or psychiatric conditions that could influence daytime sleepiness or nocturnal sleep [including dementia, hypothyroidism, narcolepsy, chronic obstructive pulmonary disease (COPD), arthritis, liver cirrhosis, or depression]; a history of insufficient habitual sleep (< 6 h per night); current or recent use of hypnotic or antidepressant medications; and engagement in shift work.

### Sample size and sampling

The sample size was calculated assuming a prevalence of EDS of 44.6% among female patients with OSA (Ulander et al., 2022). The calculation targeted 80% statistical power at a two-sided significance level of 0.05. Given 10 candidate predictor variables, the required sample size was estimated to be 226 participants.

### Outcome measures and data collection

EDS was assessed using the Thai version of the ESS (Banhiran et al., 2011), an eight-item questionnaire that rates the likelihood of dozing in daily situations (0–3 per item; total score 0–24). The Thai version has demonstrated good internal consistency, with a Cronbach's alpha coefficient of 0.87. ESS questionnaires were completed as part of routine clinical assessment on the day of the PSG. Patients with an ESS score ≥ 10 were classified as having EDS, whereas those with a score < 10 were classified as non-EDS. Baseline characteristics (age, BMI, neck circumference, and comorbidities) and polysomnographic parameters (arousal index, sleep efficiency, RDI, supine RDI, non-supine RDI, REM RDI, NREM RDI, mean oxygen saturation, minimum oxygen saturation, and time with oxygen saturation below 90% as a percentage of total sleep time [T90]) were abstracted from medical records and Level 1 PSG reports using a standardized data abstraction form.

### Polysomnography

All participants underwent in-laboratory Level 1 PSG, including both full-night and split-night studies, at the sleep laboratory of Samitivej Thonburi Hospital, Thailand. Standardized recordings included frontal, central, and occipital electroencephalography (EEG), electrooculography (EOG), submental and anterior tibialis electromyography (EMG), electrocardiography, airflow measured by an oronasal thermocouple and nasal pressure transducer, thoracoabdominal respiratory effort, snoring sensor, body position, and pulse oximetry.

Sleep staging and event scoring were conducted manually in 30-s epochs according to the American Academy of Sleep Medicine (AASM) 2012 Manual for the Scoring of Sleep and Associated Events. Apnea was defined as a reduction in airflow of at least 90% from baseline persisting for ≥10 s with continued respiratory effort. Hypopnea was defined as a decrease in airflow of at least 30% lasting for a minimum of 10 s associated with either a ≥ 3% oxygen desaturation or an EEG arousal, in accordance with AASM criteria. Respiratory effort–related arousals (RERAs) were defined as sequences of breaths lasting ≥10 s that showed increasing respiratory effort or nasal pressure signal flattening, followed by EEG arousal, but that did not meet the criteria for apnea or hypopnea. All polysomnographic recordings were scored by polysomnographic technologists who were certified sleep technologists by the Sleep Society of Thailand, with supervision provided by board-certified sleep physicians.

The RDI was calculated as the total number of obstructive apneas, hypopneas, and RERAs per hour of total sleep time. Supine and non-supine RDI were calculated using sleep duration in supine and non-supine positions, respectively. Rapid eye movement (REM) RDI and NREM RDI were calculated using time spent in REM and NREM stages, respectively. Mean and minimum oxygen saturation were derived from continuous pulse oximetry, and T90 was defined as the percentage of total sleep time spent at oxygen saturation below 90%.

According to current guidelines from the AASM, split-night protocols are recommended for their efficiency in providing both diagnostic and therapeutic interventions within a single night. At our center, polysomnographic assessments are performed in accordance with AASM criteria for the diagnosis of OSA. In split-night studies, continuous positive airway pressure (CPAP) titration was initiated when diagnostic criteria for moderate-to-severe OSA were met during at least 2 h of the diagnostic recording and a minimum of 3 h remained available for CPAP titration. For the present analysis, all respiratory indices and oxygenation parameters were derived from the diagnostic portion of the recording prior to CPAP initiation (Kapur et al., 2017). Both full-night and split-night polysomnography were included in this study to reflect clinical practice and to ensure adequate representation of disease severity, particularly among patients with moderate to severe OSA, who are more likely to undergo split-night testing in clinical settings. Prior evidence has shown that the AHI derived from the first 2–3 h of sleep strongly correlates with full-night AHI, supporting the reliability of split-night studies (Khawaja et al., 2010).

### Data analysis

All analyses were performed using Stata version 16.1 (StataCorp, College Station, TX). Only participants with complete data were included, as no missing values were observed for the variables analyzed. Participant characteristics and polysomnographic parameters were summarized using descriptive statistics. Continuous variables are presented as mean ± SD; variables that were not normally distributed were reported as median (IQR), while categorical variables are reported as frequencies and percentages. Comparisons between women with and without EDS were conducted using independent *t*-tests for continuous variables and chi-square tests for categorical variables.

Associations between individual polysomnographic parameters and EDS were assessed using multivariable logistic regression models adjusted for age, BMI, and neck circumference. The results were presented as adjusted odds ratios (aORs) and 95% confidence intervals (CIs). In addition, a four-variable polysomnographic multivariable model was constructed to evaluate the joint association of selected polysomnographic parameters with EDS and the model's discriminative ability, quantified by the area under the receiver operating characteristic curve (AUROC).

### Ethics approval and informed consent

The study was conducted in accordance with the Declaration of Helsinki, and approved by the Dhurakij Pundit University Human Research Ethics Committee (COA No. 054/67, 16 June 2025). The study was registered with the Thai Clinical Trials Registry (TCTR20250619001). Furthermore, this manuscript was prepared in accordance with the STROBE checklist for reporting observational studies. Patient consent was waived, as the study was retrospective in nature and utilized existing medical records without direct patient contact or any impact on routine clinical care.

## Results

### Baseline characteristics and polysomnographic parameters according to EDS status

**Figure 1:**
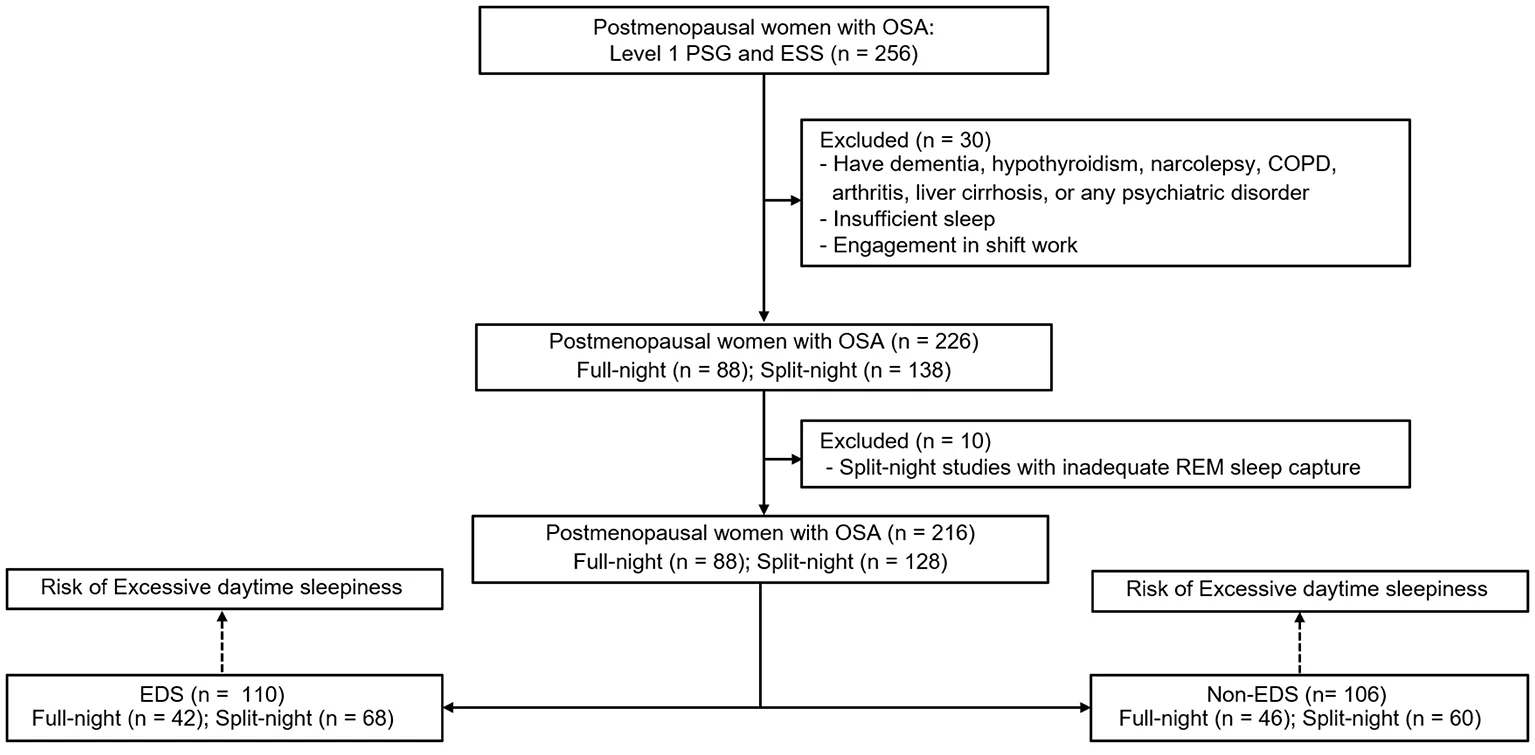
Study flow diagram.

Of 256 postmenopausal women with OSA assessed for eligibility, 30 were excluded, leaving 226 participants (88 full-night and 138 split-night polysomnographic studies). Among the split-night studies, 10 participants were excluded due to the absence of REM sleep, resulting in a final analytic sample of 216 participants (88 full-night and 128 split-night studies). Based on the ESS, 110 women (ESS ≥ 10) were classified as having EDS, whereas 106 (ESS < 10) were classified as non-EDS (Figure 1).

Baseline demographic and polysomnographic characteristics are presented in Tables 1, 2. Compared with the non-EDS group, women with EDS were younger (60.3 ± 7.9 vs. 62.6 ± 8.7 years, *p* = 0.040) and had higher BMI (26.7 ± 5.2 vs. 24.9 ± 4.5 kg/m^2^, *p* = 0.008) and neck circumference (13.2 ± 1.2 vs. 12.8 ± 1.0 inches, *p* = 0.006), whereas the prevalence of underlying diseases did not differ significantly between groups. Of the ten polysomnographic parameters evaluated (Table 2), eight differed significantly: the EDS group had higher RDI (overall, supine, non-supine, REM, and NREM), lower mean and minimum oxygen saturation, and greater T90.

**Table 1:** Demographic characteristics of postmenopausal women with OSA according to EDS status.

Characteristics	EDS (*n* = 110)	Non-EDS (*n* = 106)	*P*-value
Age (years)	60.3 ± 7.9	62.6 ± 8.7	0.040
BMI (kg/m^2^)	26.7 ± 5.2	24.9 ± 4.5	0.008
Neck circumference (inches)	13.2 ± 1.2	12.8 ± 1.0	0.006
No chronic disease	50 (45.5%)	60 (56.6%)	0.101
1 chronic disease	28 (25.5%)	16 (15.1%)	0.059
2 chronic diseases	26 (23.6%)	20 (18.9%)	0.392
3 chronic diseases	6 (5.5%)	9 (8.5%)	0.380
4 chronic diseases	0 (0%)	1 (0.9)	0.307
Diabetes mellitus	11 (10.0%)	14 (13.2%)	0.461
Hypertension	33 (30.0%)	36 (34.0%)	0.532
Dyslipidemia	45 (40.9%)	31 (29.2%)	0.073
CAD	3 (2.7%)	3 (2.8%)	0.963
Old CVA	6 (5.5%)	3 (2.8%)	0.335

Values are presented as mean ± SD or n (%), as appropriate. CAD, coronary artery disease; CVA, cerebrovascular accident.

**Table 2:** Comparison of polysomnographic parameters in postmenopausal women with OSA according to EDS status.

Parameters	EDS (*n* = 110)	Non-EDS (*n* = 106)	*P*-value
Arousal index (events/hour)	31.3 ± 15.5	30.2 ± 19.1	0.634
Sleep efficiency (%)	83.7 ± 10.9	82.8 ± 11.3	0.543
RDI (events/hour)	40.1 ± 23.5	28.3 ± 23.8	< 0.001
Supine RDI (events/hour)	47.3 ± 28.0	35.6 ± 26.8	0.002
Non-supine RDI (events/hour)	23.6 ± 26.4	14.4 ± 22.3	0.006
REM RDI (events/hour)	48.8 ± 27.6	34.5 ± 24.4	< 0.001
NREM RDI (events/hour)	37.5 ± 25.4	26.4 ± 25.1	0.002
Mean oxygen saturation (%)	94.4 ± 3.6	95.4 ± 2.7	0.018
Minimum oxygen saturation (%)	79.0 ± 11.3	83.2 ± 9.1	0.002
T90 (%)	1.0 (0.1–4.0)	0.2 (0–1.5)	0.007

Values are presented as mean ± SD; T90 is presented as median (IQR).

### Associations of polysomnographic parameters with EDS in postmenopausal women with OSA

The association between each polysomnographic parameter and EDS was examined in logistic regression models adjusted for age, BMI, and neck circumference. aORs, 95% CIs, and AUROC values are shown in Table 3. Five parameters were significantly associated with EDS (*p* < 0.05): higher RDI, supine RDI, REM RDI, and NREM RDI were associated with increased odds of EDS, whereas higher minimum oxygen saturation was associated with lower odds of EDS.

**Table 3:** Associations between individual polysomnographic parameters and EDS in postmenopausal women with OSA.

Potential prognostic factors	AUROC	aOR	95% CI	*P*-value
Arousal index (events/hour)	0.559	1.00	0.98–1.02	0.914
Sleep efficiency (%)	0.516	1.00	0.97–1.03	0.953
RDI (events/hour)	0.684	1.02	1.01–1.04	0.004
Supine RDI (events/hour)	0.637	1.01	1.00–1.03	0.019
Non-supine RDI (events/hour)	0.607	1.01	1.00–1.03	0.052
REM RDI (events/hour)	0.655	1.02	1.01–1.03	0.002
NREM RDI (events/hour)	0.661	1.02	1.00–1.03	0.011
Mean oxygen saturation (%)	0.603	0.93	0.82–1.04	0.205
Minimum oxygen saturation (%)	0.609	0.97	0.94–1.00	0.036
T90 (%)	0.606	1.02	0.97–1.07	0.512

All models were adjusted for age, BMI, and neck circumference.

### Multivariable association of polysomnographic parameters with EDS in postmenopausal women with OSA

**Figure 2:**
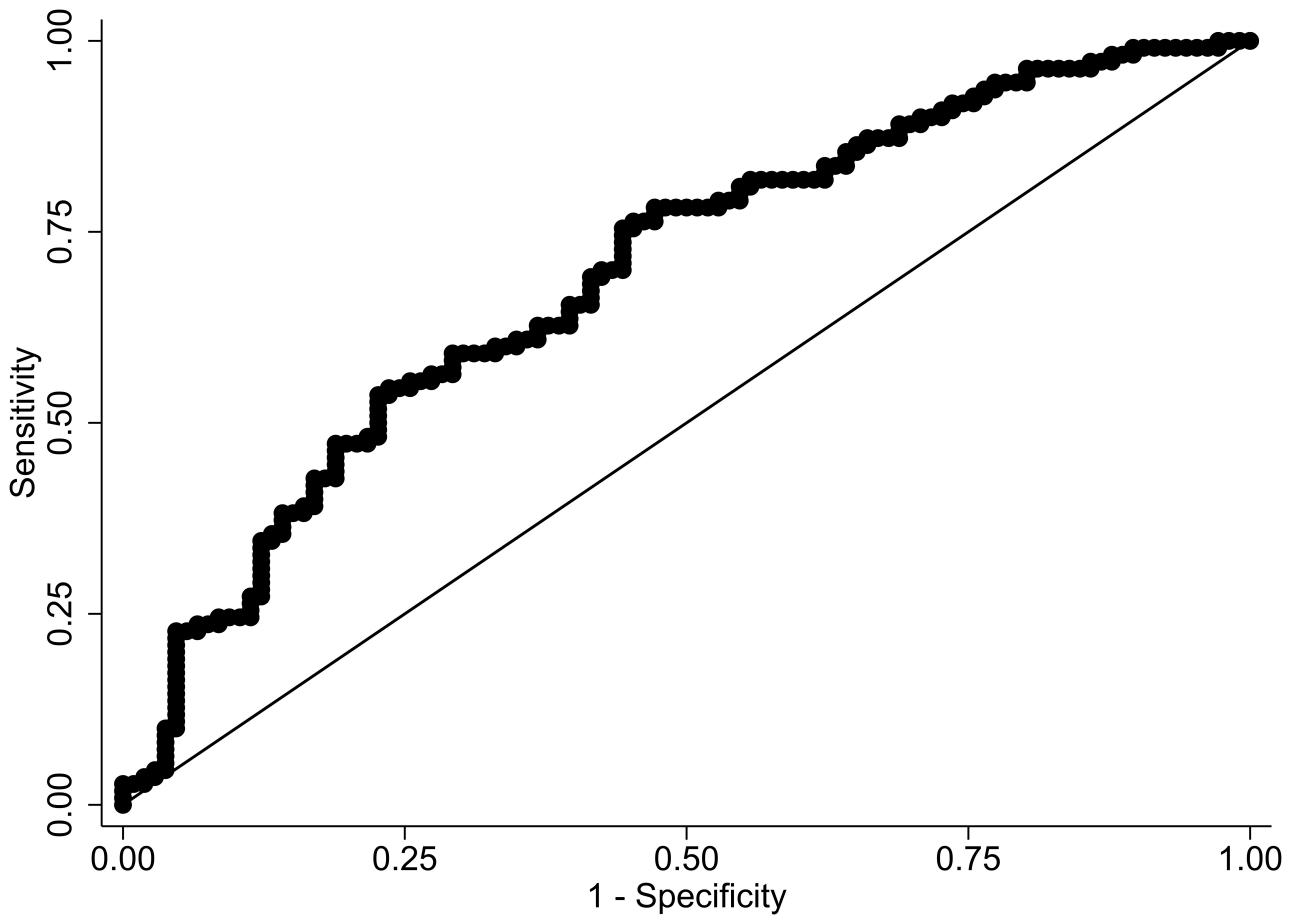
ROC curve of the multivariable model combining RDI, REM RDI, mean oxygen saturation, and minimum oxygen saturation for discriminating EDS among postmenopausal women with OSA (AUROC = 0.693).

All ten polysomnographic variables (Table 3) were initially considered as candidate predictors. A multivariable logistic regression model was developed to evaluate the joint association of selected polysomnographic parameters with EDS in postmenopausal women with OSA. Four predictors—RDI, REM RDI, mean oxygen saturation, and minimum oxygen saturation—were retained in the final model based on their univariable performance and overall model discrimination. Adding an additional predictor did not improve discriminative performance; therefore, the four-predictor model was selected because it outperformed all alternative three-predictor models and demonstrated the best overall performance among candidate multivariable models. In the final model, the aORs for EDS were 1.02 (95% CI 0.99–1.04) for RDI, 1.01 (95% CI 1.00–1.03) for REM RDI, 1.09 (95% CI 0.94–1.28) for mean oxygen saturation, and 0.98 (95% CI 0.95–1.02) for minimum oxygen saturation. The four-variable model yielded an AUROC of 0.693 (Figure 2), suggesting moderate discriminative ability. The positive predictive value (PPV) was 63.89% and a negative predictive value (NPV) was 62.04%. Multicollinearity evaluation using the variance inflation factor (VIF) showed a mean VIF of 2.54 and each variable within acceptable limits.

## Discussion

Participants who underwent split-night PSG but had no recorded REM sleep were excluded. The absence of REM sleep may be attributable to the relatively short diagnostic window (all cases had total sleep time >2 h), during which REM sleep—typically occurring later in the night—may not be captured. Prolonged REM latency and sleep fragmentation may further contribute to its absence (Kryger et al., 2010). Including these cases could bias the analysis of REM-related parameters, particularly by underestimating REM-specific disease burden. Accordingly, 10 participants were excluded, resulting in a final analytic sample of 216 participants.

The prevalence of EDS among postmenopausal women with OSA was 50.9%, which is comparable to that reported among women in the large SESAR cohort (44.6%) (Ulander et al., 2022) and falls within the range observed in newly diagnosed OSA populations (40.5–58%) (Lal et al., 2021). Postmenopausal women with EDS were younger, had higher BMI and larger neck circumference than those without EDS. These findings are consistent with previous studies reporting that obesity, central adiposity, and increased neck circumference are important determinants of OSA severity and EDS (Perger et al., 2019; Mirer et al., 2017; Kapur et al., 2017; Heinzer et al., 2018; Huang et al., 2018). However, the inverse association with age observed in this study differs from previous evidence suggesting that aging is associated with increased OSA burden and sleepiness (Lv et al., 2023). Some population-based cohorts have suggested that, beyond midlife, age-related reductions in sleep need and adaptation to chronic sleep disruption may attenuate subjective EDS despite persistent OSA (Lal et al., 2021; Berger et al., 2021; Gandhi et al., 2021). Our findings are compatible with this concept. Among postmenopausal women with established OSA, younger age within this group may reflect higher physiological sleep drive, potentially compounded by greater exposure to chronic sleep deprivation and increased vulnerability to sleep fragmentation.

Polysomnographic findings indicated that RDI (overall, supine, REM, and NREM) and minimum oxygen saturation were independently associated with EDS, whereas other parameters, such as sleep efficiency and arousal index, were not. Prior studies in OSA cohorts have similarly reported positive associations between respiratory event indices, nocturnal hypoxemia markers, and EDS, although previous studies have reported varied findings (Roure et al., 2008; Sun et al., 2012; Mediano et al., 2007; Park et al., 2018; Kainulainen et al., 2019; Hsieh et al., 2022). Our observation that minimum oxygen saturation, rather than mean oxygen saturation or T90, was most strongly related to EDS is consistent with data showing that the depth of oxygen desaturation correlates more closely with daytime sleepiness than AHI or time-based metrics alone (Sun et al., 2012; Lal et al., 2021; Park et al., 2018; Kainulainen et al., 2019; Yang et al., 2013). In contrast, several studies have reported associations between EDS and sleep efficiency or arousal index (Mediano et al., 2007; Suliman et al., 2022), whereas these associations were not evident in our study. The differing findings may be explained by differences in the characteristics of the study populations (postmenopausal women vs. mixed or predominantly male cohorts), OSA severity, and the relative contribution of hypoxemia vs. cortical arousals to subjective sleepiness.

OSA severity tends to increase during the menopausal transition, with AHI progressively worsening from perimenopause to postmenopause, independent of age and body composition. In perimenopausal women, each additional year since menopause onset has been associated with an approximately 4% increase in AHI, underscoring menopause as an important contributor to OSA burden (Mirer et al., 2017). Another study comparing women across menopausal stages has demonstrated that postmenopausal women exhibit higher AHI and REM-AHI, along with lower minimum oxygen saturation compared with women in the menopausal transition (Dugral and Ordu, 2021).

Several pathophysiological mechanisms may explain the associations between respiratory disturbance indices, nocturnal desaturation, and EDS in postmenopausal women with OSA. Menopause-related hormonal decline is an important driver of the biological changes. Declines in estrogen and progesterone levels can adversely affect upper airway physiology and disrupt sleep regulation. Progesterone has a stimulatory effect on ventilatory drive and upper airway dilator muscle activity, while estrogen is associated with sleep stability and reduced arousal susceptibility. The loss of protective hormonal effects leads to increased upper airway collapsibility, reduced neuromuscular responsiveness, and increased susceptibility to sleep-disordered breathing (Jordan et al., 2014; Lv et al., 2023; Qiu and Mateika, 2021; Antonaglia et al., 2025). In our study, women with EDS demonstrated significantly higher RDI values and more pronounced nocturnal oxygen desaturation than those without EDS, suggesting a greater burden of sleep-disordered breathing and intermittent hypoxia. Increased respiratory disturbances may contribute to sleep fragmentation and impaired sleep continuity, while oxygen desaturation may promote EDS through the physiological effects of recurrent hypoxia (Lal et al., 2021; Lv et al., 2023; Kainulainen et al., 2019; Yang et al., 2013).

Menopause is also associated with alterations in body composition, including increased central adiposity and increased BMI. Postmenopausal women tend to have greater fat deposition around the upper airway and within the thoracic cavity, thereby increasing pharyngeal collapsibility and airway resistance (Perger et al., 2019; Mirer et al., 2017; Heinzer et al., 2018; Huang et al., 2018; Hemachandra et al., 2024). However, anatomical changes alone do not fully explain the increase in OSA severity in women, suggesting that other factors, such as hormonal and functional mechanisms, may also play a complementary role in disease expression (Antonaglia et al., 2025). These structural and functional changes may be further exacerbated during specific sleep states and positions. In women, respiratory events are more likely to occur during REM sleep, a stage characterized by generalized muscle atonia and altered ventilatory control, leading to longer and more severe obstructive events with deeper oxygen desaturations (Jordan et al., 2014; Bonsignore et al., 2024; Alzoubaidi and Mokhlesi, 2016). The supine position may further reduce upper airway patency due to gravitational influences, particularly in individuals with reduced lung volumes and increased airway collapsibility (Jordan et al., 2014; Lv et al., 2023; Qiu and Mateika, 2021; Cheng et al., 2024).

These processes lead to recurrent obstructive events, intermittent hypoxemia, and sleep fragmentation, all of which are key contributors to EDS. Intermittent hypoxia, especially in cases with profound oxygen desaturation nadirs, has been associated with oxidative stress, neuroinflammation, and dysregulation of neurotransmitter systems, resulting in structural and functional impairment of wake-promoting neural circuits (Lal et al., 2021; Kainulainen et al., 2019; Yang et al., 2013; Li et al., 2014). Recurrent desaturations may also activate the sympathetic nervous system, increase arousals, and disrupt slow-wave and REM sleep, which are essential for physical restoration and cognitive function (Jordan et al., 2014; Lv et al., 2023; Kainulainen et al., 2019; Punjabi et al., 2002). This mechanistic framework is consistent with our observation that both RDI (particularly REM RDI) and minimum oxygen saturation jointly contribute to EDS risk, and supports the use of hypoxemia-focused metrics—such as nadir oxygen saturation—alongside event frequency when assessing daytime consequences of OSA in postmenopausal women.

Our four-parameter multivariable model, incorporating RDI, REM RDI, mean oxygen saturation, and minimum oxygen saturation, demonstrated moderate discrimination for identifying postmenopausal women with OSA who have EDS (AUROC 0.693). This level of performance is consistent with the discriminative ability reported in studies examining clinical and polysomnographic correlates of EDS in OSA (Garbarino et al., 2018; Jordan et al., 2014; Yang et al., 2013). From a patient-engagement perspective, given the association of EDS with increased cardiovascular, metabolic, neurocognitive, and safety-related complications (Lal et al., 2021; Gandhi et al., 2021), the polysomnographic parameters identified in this study may further inform risk communication, guide earlier initiation of CPAP therapy, and support safety counseling regarding activities requiring sustained alertness, such as driving or operating machinery (Kainulainen et al., 2019; Bonsignore et al., 2024).

Although polysomnographic variables are important correlates of EDS, they are insufficient to fully explain individual variability in EDS. Additional non-polysomnographic factors—such as low-grade systemic inflammation, metabolic dysregulation, and hormonal influences, including hormone replacement therapy—may contribute to or interact with EDS (Lal et al., 2021; Lv et al., 2023; Qiu and Mateika, 2021). Furthermore, the clinical presentation of EDS in women may differ from that in men. Women more frequently report fatigue, low energy, or non-restorative sleep rather than classical sleepiness, which may not be fully captured by the ESS questionnaire. This difference in symptom perception may lead to an underestimation of EDS and may partially explain the variability in the associations between polysomnographic parameters and EDS (Antonaglia et al., 2025). Another important consideration is the potential confounding effect of insomnia on ESS. Recent evidence suggests that insomnia severity is more strongly associated with ESS scores than polysomnographic parameters in patients with OSA. Therefore, ESS may reflect both OSA-related physiological abnormalities and coexisting insomnia symptoms. Because insomnia was not systematically assessed in our retrospective study, residual confounding cannot be excluded when interpreting our findings. Future prospective studies should incorporate standardized insomnia assessment to better distinguish the independent contributions of insomnia and OSA to EDS (Gouveris et al., 2025).

The strengths of this study include a focused sample of postmenopausal women with OSA, the use of standardized Level 1 PSG in a single sleep laboratory, the application of a validated Thai version of the ESS, and the use of multivariable modeling to evaluate polysomnographic parameters (Banhiran et al., 2011; Kapur et al., 2017). However, several limitations should be acknowledged. The retrospective design limited the availability of additional clinical information, including duration since menopause, hormone replacement therapy use, and beta-blocker use, which were not systematically collected (Perger et al., 2019; Mirer et al., 2017; Huang et al., 2018; Qiu and Mateika, 2021). The inclusion of split-night studies may have led to underestimation of REM-specific and position-dependent respiratory indices in some patients. However, these studies were retained to ensure adequate representation of individuals with moderate to severe OSA, as their exclusion would have substantially reduced the number of patients with more severe disease. Furthermore, previous studies have demonstrated that the AHI derived from the initial 2–3 h of sleep during split-night polysomnography correlates strongly with the full-night AHI, supporting the validity of split-night studies for OSA diagnosis and severity classification (Khawaja et al., 2010). Participants without recorded REM sleep were excluded, further reducing the sample size and potentially affecting statistical power. Finally, the model was developed using a derivation cohort and demonstrated only moderate discriminative ability, which may limit its utility for individual-level clinical decision-making. External validation in independent populations is required to confirm its performance and clinical applicability.

## Conclusions

EDS was more common among postmenopausal women with OSA who had a greater respiratory burden and more severe nocturnal hypoxemia. RDI, supine RDI, REM RDI, and NREM RDI were positively associated with EDS, whereas minimum oxygen saturation was inversely associated with EDS. These polysomnographic parameters may help identify postmenopausal women with OSA who are at increased risk of clinically significant EDS. Future studies incorporating more comprehensive, multidimensional data may further improve model performance and enhance clinical applicability.

## Data Availability

The datasets presented in this article are not readily available due to patient privacy and institutional restrictions. Requests to access the datasets should be directed to the corresponding author and are subject to approval by Samitivej Thonburi Hospital. Requests to access the datasets should be directed to Mart.mai@dpu.ac.th.
