# Supplementary material for: Polysomnographic characteristics of excessive daytime sleepiness in postmenopausal women with obstructive sleep apnea
Source: Front Sleep. 2026 Aug 25;5:1853970. doi: 10.3389/frsle.2026.1853970 (PMC13549838; doi:10.3389/frsle.2026.1853970)
Supplement: Supplementary file 1 [file Table_1.docx]

Supplementary Material

**Polysomnographic Characteristics of Excessive Daytime Sleepiness in Postmenopausal Women with Obstructive Sleep Apnea**

**Thanida Manitchotpisit^1^, Pansak Sugkraroek^1^, Charnsiri Segsarnviriya^2^, Naricha Chirakalwasan^3,4^ and Mart Maiprasert^1,*^**

^1^Department of Anti-Aging and Regenerative Medicine, College of Integrative Medicine, Dhurakij Pundit University, Bangkok, Thailand

^2^Department of Otorhinolaryngology, Samitivej Thonburi Hospital, Bangkok, Thailand

^3^Division of Pulmonary and Critical Care Medicine, Department of Medicine, Faculty of Medicine, Chulalongkorn University, Bangkok, Thailand

^4^Excellence Center for Sleep Disorders, King Chulalongkorn Memorial Hospital, Thai Red Cross Society, Bangkok, Thailand

*** Correspondence:**

Mart Maiprasert

Mart.mai@dpu.ac.th

# Table S1. AUROC of multivariable polysomnographic models using combinations of 2–5 prognostic factors for predicting EDS in postmenopausal women with OSA; the top three AUROC-performing models are presented for each number of predictors.

| No. of predictors | Model | AUROC |
| --- | --- | --- |
| 2 predictors | REM RDI, mean oxygen saturation | 0.671 |
|  | REM RDI, minimum oxygen saturation | 0.677 |
|  | RDI, minimum oxygen saturation | 0.683 |
|  | RDI, REM RDI | 0.688 |
|  | RDI, mean oxygen saturation | 0.689 |
| 3 predictors | RDI, REM RDI, minimum oxygen saturation | 0.685 |
|  | RDI, REM RDI, supine RDI | 0.687 |
|  | RDI, minimum oxygen saturation, mean oxygen saturation | 0.689 |
|  | RDI, REM RDI, mean oxygen saturation | 0.691 |
| 4 predictors | RDI, REM RDI, supine RDI, minimum oxygen saturation | 0.685 |
|  | RDI, REM RDI, supine RDI, mean oxygen saturation | 0.691 |
|  | RDI, REM RDI, non-supine RDI, minimum oxygen saturation | 0.686 |
|  | RDI, REM RDI, minimum oxygen saturation, mean oxygen saturation | 0.693 |
| 5 predictors | RDI, REM RDI, mean oxygen saturation, minimum oxygen saturation, non-supine RDI | 0.692 |
|  | RDI, REM RDI, mean oxygen saturation, minimum oxygen saturation, supine RDI | 0.693 |
|  | RDI, REM RDI, mean oxygen saturation, minimum oxygen saturation, NREM RDI | 0.692 |
|  | RDI, REM RDI, mean oxygen saturation, minimum oxygen saturation, efficiency | 0.691 |

# Table S2. Variance Inflation Factors (VIF) and tolerance values for predictors included in the final model

| Variable | VIF | 1/VIF (tolerance) |
| --- | --- | --- |
| RDI | 3.20 | 0.31 |
| REM RDI | 2.41 | 0.42 |
| mean oxygen saturation | 2.66 | 0.38 |
| minimum oxygen saturation | 1.91 | 0.52 |
| mean VIF | 2.54 |  |

# Table S3. Sensitivity analyses of the association between REM RDI and EDS

| Analysis for REM RDI | EDS (n) | Non-EDS (n) | aOR | 95% CI | P value |
| --- | --- | --- | --- | --- | --- |
| All participants | 110 | 106 | 1.02 | 1.01–1.03 | 0.002 |
| Full-night PSG | 42 | 46 | 1.03 | 1.01–1.05 | 0.011 |
| Split-night PSG | 68 | 60 | 1.02 | 1.00–1.04 | 0.030 |
